# Stiffening hydrogels for investigating the dynamics of hepatic stellate cell mechanotransduction during myofibroblast activation

**DOI:** 10.1038/srep21387

**Published:** 2016-02-24

**Authors:** Steven R. Caliari, Maryna Perepelyuk, Brian D. Cosgrove, Shannon J. Tsai, Gi Yun Lee, Robert L. Mauck, Rebecca G. Wells, Jason A. Burdick

**Affiliations:** 1 Department of Bioengineering, University of Pennsylvania, Philadelphia, PA 19104, USA; 2 Department of Medicine, University of Pennsylvania, Philadelphia, PA 19104, USA; 3 Department of Orthopedic Surgery, University of Pennsylvania, Philadelphia, PA 19104, USA.

## Abstract

Tissue fibrosis contributes to nearly half of all deaths in the developed world and is characterized by progressive matrix stiffening. Despite this, nearly all *in vitro* disease models are mechanically static. Here, we used visible light-mediated stiffening hydrogels to investigate cell mechanotransduction in a disease-relevant system. Primary hepatic stellate cell-seeded hydrogels stiffened *in situ* at later time points (following a recovery phase post-isolation) displayed accelerated signaling kinetics of both early (Yes-associated protein/Transcriptional coactivator with PDZ-binding motif, YAP/TAZ) and late (alpha-smooth muscle actin, α-SMA) markers of myofibroblast differentiation, resulting in a time course similar to observed *in vivo* activation dynamics. We further validated this system by showing that α-SMA inhibition following substrate stiffening resulted in attenuated stellate cell activation, with reduced YAP/TAZ nuclear shuttling and traction force generation. Together, these data suggest that stiffening hydrogels may be more faithful models for studying myofibroblast activation than static substrates and could inform the development of disease therapeutics.

Tissue fibrosis contributes to nearly half of all deaths in the developed world^1^. Fibrosis develops in response to a wide range of injuries in many tissues, including the pancreas, lung, kidney, and liver^2^,^3^. The differentiation of pericytes and fibroblasts to fibrogenic myofibroblasts is central to the development of fibrosis and results in aberrant extracellular matrix (ECM) synthesis and remodeling, which in turn leads to tissue stiffening and loss of organ function. Hepatic stellate cells are the primary source of myofibroblasts in liver fibrosis^4^ and primary liver cancer (often associated with fibrosis), which together account for over 1.7 million deaths annually worldwide^5^.

There is a growing appreciation for the role that local mechanical properties play in the initiation, development, and progression of pathologies such as liver fibrosis, especially in light of recent evidence that tissue stiffening, which is generally regarded as an outcome of disease, may be a significant contributing factor in the development of fibrosis and in tumor formation^6^,^7^,^8^. Hydrogel biomaterials are increasingly being applied as model microenvironments to probe cellular behavior with seminal studies defining the roles of substrate stiffness^9^, geometry^10^, and degradability^11^ in regulating cell fate. The role of mechanics in stellate cell differentiation has been investigated using several hydrogel platforms to show that hepatic stellate cells cultured on compliant (elastic modulus (*E*) or stiffness <3 kPa) substrates with stiffness levels similar to healthy liver remain phenotypically quiescent, while stellate cells cultured on stiffer substrates with stiffness levels similar to diseased liver (*E* > 20 kPa) undergo myofibroblast differentiation^12^,^13^,^14^.

Despite this, there is a paucity of *in vitro* model systems that can recapitulate the temporal dynamics of mechanics during disease; all previous studies of hepatic stellate cells during myofibroblast activation have occurred on mechanically static substrates. Unfortunately, these static materials may not replicate the changes observed *in vivo*, as primary cells are digested from tissue and directly seeded onto substrates rather than experiencing gradual or delayed stiffening. Although hepatic stellate cells *in vivo* up-regulate fibrogenic proteins such as connective tissue growth factor and β-platelet-derived growth factor receptor within several hours following fibrosis induction^15^,^16^, many freshly isolated primary cells (including stellate cells) show altered expression of integrins and other surface proteins^17^,^18^ that may attenuate their response to early mechanical stimuli, highlighting the importance of the timing of mechanical signals for *in vitro* disease models. Additionally, as new molecular targets are identified, it is important to understand them in dynamic systems that are relevant to the *in vivo* setting. For example, the Hippo-related transcriptional co-activators Yes-associated protein/Transcriptional coactivator with PDZ-binding motif (YAP/TAZ) have been identified as important mechanosensing regulators involved in stem cell differentiation^19^,^20^ and were recently shown to be responsive to dynamic mechanical loading^21^. However, the role of YAP/TAZ in the mechanically-driven progression of fibrosis has not been investigated in the context of changing mechanics^16^,^22^.

Various stiffening systems have been used to investigate stem cell fate decisions^23^, cell spreading^24^, cardiomyocyte maturation^25^, and myofibroblast phenotype in heart disease^26^. Collectively, these studies suggest that both the magnitude and the timing of mechanical signals are important for investigating mechanotransduction signaling dynamics in complex cell behaviors such as stem cell differentiation and myofibroblast activation. However, previous approaches do not offer precise temporal control of mechanical signals^25^ or are limited by their use of ultraviolet (UV) light^23^,^26^, which may be harmful to some cell types (including stellate cells^27^). In this work, we aimed to develop a hydrogel system amenable to *in situ* stiffening in the presence of sensitive primary cell types like hepatic stellate cells to investigate how stiffening events influence the dynamics of both early (YAP/TAZ nuclear translocation) and late (actin cytoskeleton maturation) events in stellate cell myofibroblast differentiation.

## Results

### Visible light-mediated secondary crosslinking enables stiffening of MeHA hydrogels

**Figure 1:**
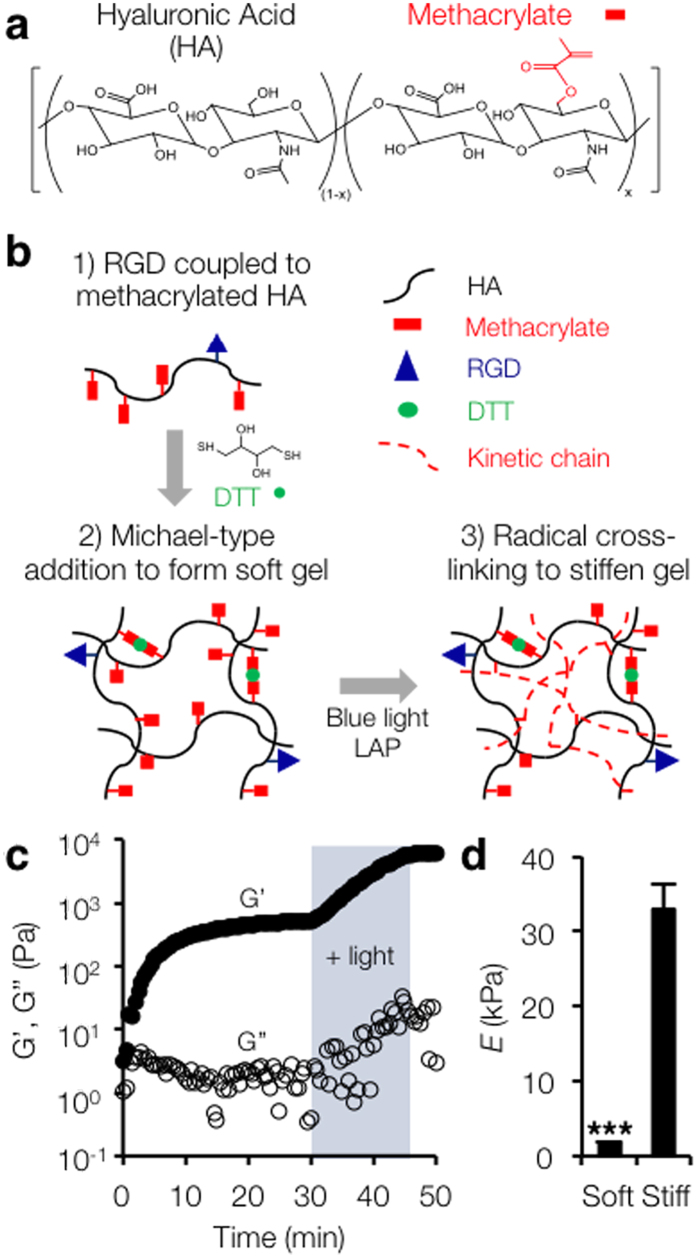
Visible-light mediated stiffening of MeHA gels. (a) Hyaluronic acid was modified with methacrylates to enable both addition and radical crosslinking. (**b**) RGD-containing peptide was coupled to MeHA followed by an initial Michael-type addition reaction to form a soft gel. Radical polymerization in the presence of visible blue light and the photoinitiator LAP enabled substrate stiffening. (**c**) Rheology profile showing stiffening of MeHA in response to blue light (3 mW/cm^2^, 15 min, shaded) in the presence of LAP (6.6 mM). Closed circles represent storage modulus (G’), open circles represent loss modulus (G”). (**d**) AFM measurements of both soft and stiff (blue light: 10 mW/cm^2^, 5 min, LAP: 6.6 mM) hydrogel elastic moduli (*n* = 3 gels per group, error bars represent s.e.m.). ***P < 0.001.

We observed that UV irradiation significantly reduced hepatic stellate cell viability and increased nuclear p53 accumulation when compared to visible (blue) light or no irradiation (Supplementary Fig. 1). Based on these results, we conceived of a scheme to fabricate stiffening hydrogels from methacrylated hyaluronic acid (MeHA) (Fig. 1a). This approach combined an initial Michael-type addition reaction to form a soft gel followed by visible light-mediated radical polymerization of unreacted methacrylates at a user-defined time point to stiffen the substrate (Fig. 1b). Although we considered several initiators (e.g., Eosin Y, Supplementary Fig. 2), lithium acylphosphinate (LAP)^28^ was identified as the most promising since there was no reduction in hepatic stellate cell viability even at high LAP concentrations (6.6 mM) or with combined LAP (6.6 mM) and visible blue light exposure (5 min, 10 mW cm^−2^, peak wavelength ~470 nm) (Supplementary Fig. 3). The combination of blue light exposure and LAP for secondary crosslinking enabled significant substrate stiffening as measured by rheometry (Fig. 1c) and atomic force microscopy (Fig. 1d). Using this approach, we fabricated both “soft” substrates (*E* = 1.75 ± 0.02 kPa, corresponding to healthy liver stiffness) and secondary crosslinked “stiff” substrates (*E* = 33.0 ± 3.2 kPa, fibrotic liver stiffness).

### Hepatic stellate cells spread and assume myofibroblast morphology in response to *in situ* stiffening

**Figure 2:**
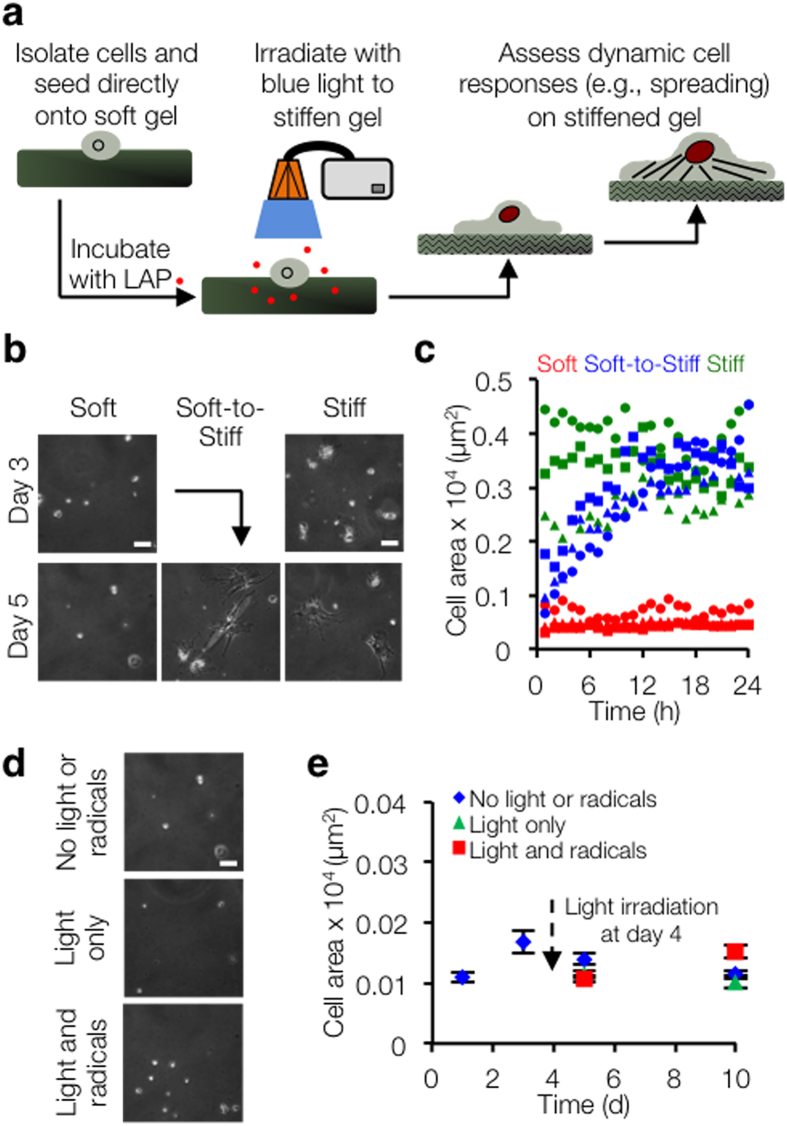
Hepatic stellate cells spread and assume myofibroblast morphology in response to *in situ* stiffening. (**a**) Schematic of *in situ* stiffening process. (**b**) Phase contrast images of stellate cells on soft and stiff static substrates, as well as a dynamically stiffened soft-to-stiff substrate (*in situ* stiffening performed on day 4). Scale bars: 50 μm. (**c**) Representative stellate cell spread area quantification over 24 h time lapse following *in situ* stiffening on day 4 (*n* = 3 cells per group, each shape represents a single cell whose spread area was monitored every 15 min). Spread areas are relatively constant in soft and stiff static groups, but increase steadily in soft-to-stiff group, especially during the first 12 h. (**d**) To ensure that stellate cells were spreading in response to stiffness and not free radical generation, soft MeHA gels were fabricated where the remaining methacrylates were capped with a thiol, so that in the presence of light and initiator free radicals would be generated but no secondary crosslinking (stiffening) would occur. Representative phase contrast images showed no differences in cell spread areas between groups. Scale bars: 50 μm. (**e**) Quantification of cell area confirmed no differences in spread area between groups (*n* > 22 cells per group per time point, error bars represent s.e.m.).

Using this dynamic hydrogel system, we investigated stellate cell response to *in situ* secondary crosslinking (Fig. 2a). Hepatic stellate cells seeded on soft substrates remained rounded and retained lipid droplets, whereas stellate cells cultured on stiff substrates spread gradually over several days, lost their lipid droplets, and assumed a myofibroblast-like morphology (Fig. 2b). Meanwhile, stellate cells initially cultured on soft gels and subjected to *in situ* secondary crosslinking responded rapidly (~18–24 h) to the change in substrate stiffness, resembling the morphology of cells cultured on the stiff static substrates (Fig. 2b,c, Supplementary Movie 1). To ensure that the observed cell morphology results were due to changes in substrate stiffness and not generation of free radicals^29^, soft MeHA gels were fabricated where the remaining methacrylates were capped with a thiol, so that light exposure in the presence of a photoinitiator led to free radical generation but no increased crosslinking. No differences in hydrogel elastic modulus or cell spread area were observed when compared to cells on soft hydrogels, indicating that radical generation did not induce stellate cell spreading (Fig. 2d,e, Supplementary Fig. 4).

### Delayed stiffening promotes more rapid YAP/TAZ nuclear translocation and α-SMA stress fiber assembly

We hypothesized that the gradual cell spreading observed over several days on stiff substrates (Supplementary Fig. 5) was due to a post-isolation recovery phase where stellate cell mechanosensing was attenuated. To investigate this, we stiffened gels at either an early pre-recovery (day 1) or later post-recovery (day 6) time point and assessed stellate cell spreading over 14 total days of culture compared to soft and stiff static gels. Stellate cell spread area for early stiffening (day 1) largely mirrored trends observed for the static stiff hydrogels. Interestingly, stellate cells were observed to spread more rapidly following later stiffening (day 6) and “catch up” to early stiffening groups within 48 h (Supplementary Figs 5 and 6). AFM assessment indicated that all stiffened substrates had similar elastic moduli after 14 days, regardless of stiffening time point (Supplementary Fig. 7). While previous studies showed that hepatic stellate cells spread gradually over several days when plated onto stiff culture substrates^14^,^30^, here we show that following a culture period of several days in a soft environment that allows recovered expression of surface proteins such as platelet-derived growth factor receptor beta (β-PDGFR) and β1 integrin (Supplementary Fig. 8), stellate cells spread and activate much more rapidly in response to a step increase in stiffness.

**Figure 3:**
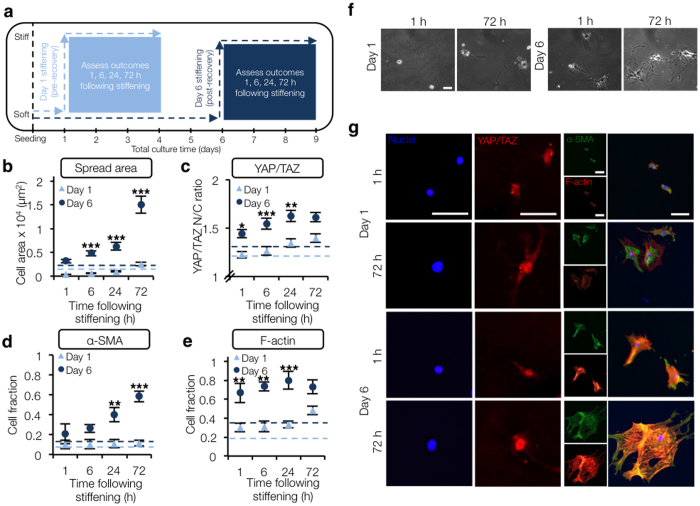
Delayed stiffening promotes more rapid YAP/TAZ nuclear translocation and α-SMA stress fiber assembly. (**a**) Schematic of experimental design. (**b**) Quantification of stellate cell spread area (*n* > 12 cells per group), (**c**) YAP/TAZ nuclear to cytoplasmic intensity ratio (*n* > 29 cells per group), (**d**) cell fraction displaying organized α-SMA stress fibers (*n* > 20 cells per group), (**e**) and cell fraction displaying organized F-actin stress fibers (*n* > 20 cells per group) 1, 6, 24, or 72 h following either early (Day 1) or later (Day 6) stiffening (error bars represent s.e.m.). (**f**) Representative phase contrast images of cells 1 or 72 h following stiffening at either day 1 or 6. (**g**) Representative immunostaining showing more rapid YAP/TAZ nuclear translocation and α-SMA stress fiber assembly at later stiffening time point (day 6). While YAP/TAZ nuclear localization was more evident 72 h following earlier stiffening (day 1), α-SMA staining was still diffuse and did not co-localize with F-actin stress fibers. In contrast, within 1 h following later stiffening (day 6) YAP/TAZ nuclear translocation was present and 72 h following stiffening the majority of stellate cells displayed α-SMA stress fibers. Representative immunostaining for all experimental groups is presented in the Supplementary Information. Dashed lines represent levels on soft gels at Day 1 (light blue) or Day 6 (dark blue). *P < 0.05, **P < 0.01, ***P < 0.001 where comparisons shown in figure are between day 1 and day 6 results for each post-stiffening time point (e.g., in panel (**c**) the day 6 YAP/TAZ nuclear intensity ratio 1 h post-stiffening is significantly greater than day 1 YAP/TAZ intensity 1 h post-stiffening, *P* < 0.05). Scale bars: 50 μm.

We next performed a series of experiments to elucidate the temporal dynamics of mechanotransduction events immediately following stiffening at either an early (day 1) or later (day 6) post-isolation recovery time point. Spread area, YAP/TAZ translocation (early marker), α-SMA expression (late marker), and F-actin organization were evaluated 1–72 h following stiffening. Results were compared to static soft gel controls (dashed lines, Fig. 3). No significant differences were observed between the static soft day 1 and day 6 controls (dashed lines) for any of the measured outcomes. YAP/TAZ nuclear intensity gradually increased following both early and late stiffening, although this change was more rapid following later stiffening with YAP/TAZ nuclear intensity 1 h after later stiffening roughly equivalent to 72 h following earlier stiffening (Fig. 3c,g, Supplementary Figs 9 and 10). While significantly higher YAP/TAZ nuclear intensity and F-actin stress fiber organization were observed following later stiffening within 1 h (Fig. 3c,e,g, Supplementary Figs 9 and 10), myofibroblast maturation as measured by α-SMA stress fiber organization lagged behind both of these metrics (Fig. 3d,g, Supplementary Figs 9 and 10). Most stellate cells displayed diffuse α-SMA staining even 72 h following early stiffening, while α-SMA stress fiber organization gradually increased after later stiffening and was significantly higher at the 24–72 h time points compared to earlier stiffening. When these experiments were extended to 14 days of total culture time, stellate cells on all stiffened substrates (static stiff, early (day 1) and late (day 6) stiffening) showed significantly elevated levels of YAP/TAZ nuclear intensity, F-actin (and more specifically α-SMA) stress fiber formation, and fibrogenic gene expression of *Acta2* and *Col1a1* (encoding for α-SMA and type I collagen, respectively) compared to stellate cells on soft gels (Supplementary Figs 11 and 12).

### Specific inhibition of α-SMA polymerization results in reduced YAP/TAZ nuclear shuttling and traction force generation

**Figure 4:**
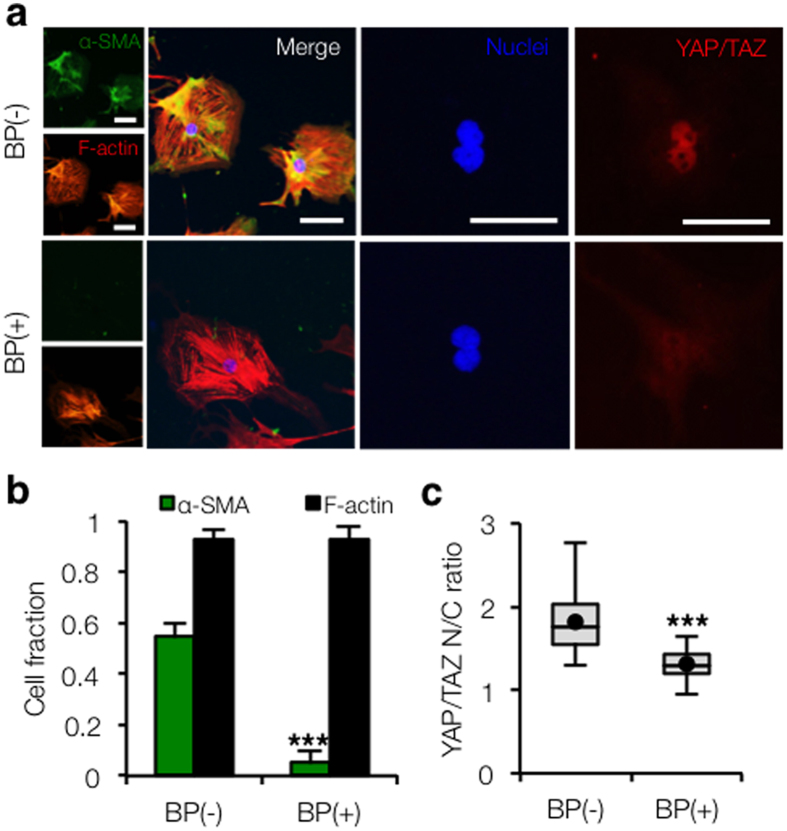
Blocking α-SMA polymerization results in reduced YAP/TAZ nuclear shuttling. (**a**) Stellate cells were seeded on soft gels that were stiffened after 3 days and cultured in normal media (BP(−)) or media supplemented with α-SMA blocking peptide (25 μg/mL, BP(+)) for an additional 4 days. The blocking peptide almost completely abrogated α-SMA expression without impacting other filamentous actin, but reduced nuclear YAP/TAZ accumulation. Scale bars: 50 μm. (**b**) Immunostaining quantification indicates that α-SMA blocking does not affect the fraction of cells displaying F-actin stress fibers (*n* > 30 cells per group, error bars represent s.e.m.). (**c**) Tukey box plot of YAP/TAZ nuclear to cytoplasmic intensity ratio demonstrated significant reduction in YAP/TAZ nuclear localization in BP(+) group (*n* > 30 cells per group). ***P < 0.001.

**Figure 5:**
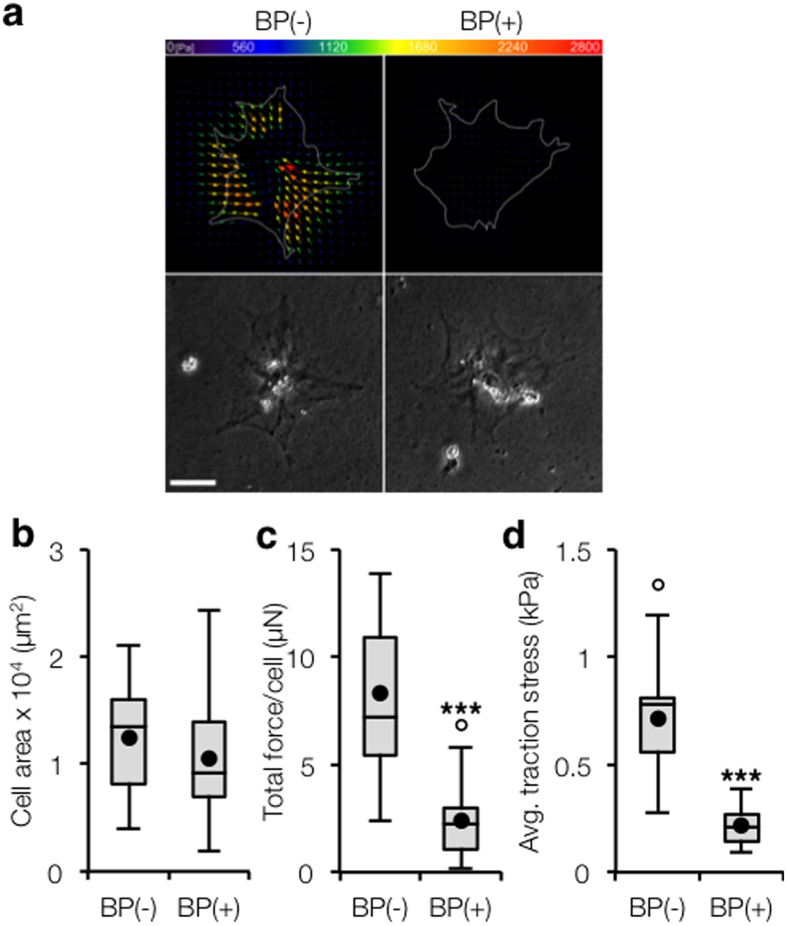
Inhibiting α-SMA does not alter stellate cell spread area but significantly reduces traction force generation. (**a**) Representative traction force vector maps and phase contrast images illustrating similar spreading but stronger tractions in BP(−) group. Scale bar: 50 μm. (**b**) Tukey box plot quantification of cell spread area (*n* > 12 cells per group). (**c**) Tukey box plot quantification of total force generated per cell (*n* > 12 cells per group). (**d**) Tukey box plot quantification of average traction stress (*n* > 12 cells per group). ***P < 0.001.

We used our dynamic stiffening system to study the connection between early YAP/TAZ -mediated mechanosensing events and later markers of myofibroblast maturation, such as α-SMA organization. We cultured stellate cells on hydrogels that were stiffened after three days of recovery and then supplemented with an α-SMA blocking peptide (BP(+))^31^ and compared outcomes to a non-treated control (BP(−)). Inhibiting α-SMA resulted in nearly complete abrogation of α-SMA staining without altering stress fiber formation of other F-actin isoforms (Fig. 4a,b). However, α-SMA blockade resulted in significantly reduced YAP/TAZ nuclear intensity (*P* < 0.001, Fig 4b,c). We hypothesized that the decrease in YAP/TAZ nuclear translocation was due to reduced mechanical feedback from α-SMA-mediated contractility. Although hepatic stellate cells are known to be sensitive to mechanical signals and are highly contractile during disease progression, to our knowledge no previous studies have quantified the traction forces exerted by these cells. Here, we used traction force microscopy (TFM) to show that treatment of stellate cells with the blocking peptide did not alter spread area but resulted in significantly decreased total force generation and average traction stresses ((BP(−) ~ 700 Pa, BP(+) ~ 200 Pa, *P* < 0.001, Fig. 5).

## Discussion

In this work we developed a cytocompatible method to fabricate *in situ* stiffening hydrogels in order to recapitulate changes in mechanical signaling that occur at the onset and during the progression of diseases such as liver fibrosis. This is an irreversible technique, where the magnitude and timing of the stiffening process are altered with light exposure parameters. Hydrogels were modified with an RGD peptide for cell adhesion; however, a range of ECM proteins and peptides could be investigated in this system. We then showed that hepatic stellate cells activated more quickly in response to delayed post-recovery stiffening in a manner reminiscent of *in vivo* activation compared to early stiffening. We further demonstrated the unique power of this system to show that inhibiting α-SMA polymerization following substrate stiffening resulted in reduced YAP/TAZ nuclear localization and traction force generation. Collectively, these data suggest that stiffening hydrogels may be superior to static culture substrates for studying the dynamics of mechanotransduction in disease and could inform the identification of targets for therapeutic intervention.

We previously developed an *in situ* stiffening hydrogel system that relied on UV light irradiation to introduce secondary crosslinking into hydrogels^23^. Although this system had many salient features towards our goals here, previous reports indicated that hepatic stellate cells are sensitive to UV light^27^, possibly due to the presence of vitamin A-containing lipid droplets^32^ in quiescent stellate cells. As an alternative approach, we demonstrated that visible light-mediated secondary crosslinking resulted in a pathologically-relevant increase in hydrogel stiffness without harming stellate cell viability.

Harnessing the power of this mechanically dynamic model system, we hypothesized that not only the stiffening event itself, but also the timing of stiffening would affect stellate cell myofibroblast phenotype as manifested in both early (YAP/TAZ) and late (α-SMA) markers. YAP/TAZ are Hippo-related transcriptional co-activators that primarily interact with TEAD-related transcription factors to regulate gene expression in a wide range of downstream processes. Although the involvement of YAP/TAZ in the (dys)regulation of organ growth during development and disease (e.g., cancer) is well-characterized^33^, its prominent role in the progression of liver fibrosis has only recently been elucidated^16^. Numerous YAP/TAZ target genes such as connective tissue growth factor (*Ctgf*) promote fibrotic responses, including increased α-SMA expression^34^, a hallmark of the myofibroblast phenotype. We showed that later stiffening within the first week of culture resulted in accelerated stellate cell spreading and α-SMA stress fiber formation, mediated by quicker YAP/TAZ nuclear translocation.

The more rapid activation observed following later stiffening is consistent with hepatic stellate cell activation kinetics *in vivo* where YAP/TAZ target genes such as *Ctgf* as well as fibrogenic proteins like β-PDGFR are up-regulated within several hours of injury^15^,^16^. The difference in signaling kinetics observed between early and late stiffening might be due to a post-isolation recovery phase that initially retards the ability of stellate cells to respond to mechanical inputs in a pathologically-relevant manner. Hepatic stellate cell gene expression profiles in cultured activated cells (including various surface proteins and cytoskeletal components) are notably different than *in vivo* activated stellate cells^18^. Although the simplified microenvironment for *in vitro* activation likely accounts for some of these differences, expression of these markers may also be skewed by protease-dependent cell isolation procedures. Indeed, we observed significantly increased expression of β1 integrin in stellate cells after 6 days culture on soft gels compared to freshly isolated cells, as well as a return to basal levels of β-PDGR following a recovery period of several days. Critically, the observed differences in signaling kinetics following cell isolation and subsequent substrate stiffening would not have been possible with conventional cell culture platforms. Previous work examining stellate cell activation on soft and stiff supports highlighted the role of mechanics and suggested that culture on soft and physiologically stiff substrates, rather than on plastic, is optimal for understanding normal stellate cell function and activation. The data presented here further suggest that culture on standard plastic supports misrepresents the kinetics of activation, and that optimal cell culture requires a post-isolation recovery period as well as mechanically physiologic substrates. Stiffening hydrogels, which address both requirements, could be ideal *in vitro* models for studying stiffness-mediated events in development and disease, including although not limited to the timing and relevance of α-SMA expression.

Collectively, our findings indicate that while initial stellate cell culture on soft substrates suppresses numerous canonical indicators of myofibroblast phenotype, including spreading and α-SMA stress fiber organization, stellate cells may still be primed for quicker activation as they recover from the isolation procedure. Despite a previous report on the mechanical memory of lung fibroblasts that found extended (~2 week) culture on soft substrates protected against stiffness-mediated myofibroblast activation^35^, our data are supported by a study of hepatic stellate cells cultured on Matrigel that showed shorter-term culture (~1 week) did not prevent activation^36^. It is also important to note that in the aforementioned studies cells were treated with proteases (similar to procedures during cell isolation) and moved to different substrates, likely altering the mechanoresponsive timeline^35^,^36^. Our system is uniquely positioned to allow cells to recover post-isolation without any exposure to aberrant mechanical signals (e.g., tissue culture plastic), and then introduce a pathologically-relevant mechanical input at a user-defined time point. Our results suggest that this approach may more faithfully capture the dynamics of mechanotransduction signaling events in disease compared to conventional static culture substrates. More broadly, our results may have implications for other applications involving the use of soft and/or stiff culture substrates as tools to prevent or guide specific differentiation fates.

Finally, we applied our mechanically dynamic hydrogel system to demonstrate that blocking α-SMA polymerization following substrate stiffening results in reduced YAP/TAZ nuclear localization and traction force generation, indicating that α-SMA contractility is critical for YAP/TAZ -mediated mechanotransduction during myofibroblast maturation. Importantly, we believe that this could only be investigated with a system such as that developed here. In these experiments, the stiffening time was less important as cells undergo activation across the range of stiffening timepoints and we chose an intermediate recovery time (3 days) prior to stiffening for investigation. This was partly to ensure that the hydrogel properties were unchanged at the time traction studies were performed. While previous studies demonstrated that α-SMA inhibition resulted in reduced contractility, motility, and Erk1/2 activity in lung^31^ and liver^37^ myofibroblasts, to our knowledge the connection between α-SMA contractility and YAP/TAZ activity in myofibroblast activation has not been made. The observed reduction in traction force generation is functionally important since activated stellate cells *in vivo* display traction force-dependent increases in motility and contractility that lead to restricted portal blood flow and permit the release of latent ECM-sequestered fibrogenic cytokines such as TGF-β^38^. Well-defined cell culture systems such as the one developed here provide platforms for improving our understanding of how mechanosensitive cells like hepatic stellate cells respond to dynamic changes in their environment and could inform the development of disease therapeutics (e.g., inhibition of YAP/TAZ translocation or α-SMA organization).

## Methods

### MeHA synthesis

Hydrogels were formed from methacrylated hyaluronic acid as previously reported^39^. 2 wt% sodium hyaluronate (Lifecore, 75 kDa) in deionized water was reacted with methacrylic anhydride (Sigma, 5.6 mL per g HA) on ice at pH 8.0–9.5 for 6 h. The solution was dialyzed at room temperature in deionized water (SpectraPor, 6–8 kDa molecular weight cutoff) for 7 d, and then lyophilized. The degree of modification was ~90% as measured by ^1^H NMR (Bruker, Supplementary Fig. 13).

### MeHA hydrogel fabrication

The adhesion ligand RGD (GCGYG*RGD*SPG, GenScript) was coupled to methacrylates on HA via a Michael-type addition reaction to enable cell attachment. MeHA and RGD peptide were incubated under shaking for 45 min at room temperature in 0.2 M triethanolamine (TEOA, Sigma) buffer at pH 9 at ratios to obtain a final RGD concentration in hydrogels of 1 mM. Soft MeHA hydrogels were also formed by Michael-type addition. 3 wt% RGD-modified MeHA was crosslinked with dithiothreitol (DTT, Sigma) at pH 9. Hydrogel precursor solution (70 μL) was pipetted into polydimethylsiloxane molds (PDMS, Dow Corning, 18 × 18 × 0.15 mm) and covered with methacrylated glass coverslips^40^ for 3 h at room temperature to form a hydrogel film attached to the functionalized coverslip. RGD functionalization and crosslinking of soft hydrogels only consumes ~20% of available methacrylate groups, leaving ample groups available for secondary crosslinking. For TFM experiments, 0.2 μm diameter fluorescent beads (Invitrogen) were mixed with hydrogel precursor solution at 1% v/v prior to gelation.

### LAP synthesis

Lithium acylphosphinate (LAP) was synthesized as previously described^28^,^41^. Briefly, equimolar amounts of dimethyl phenylphosphonite (Sigma) and 2,4,6-trimethylbenzoyl chloride (Sigma) were reacted for 18 h under constant stirring and nitrogen at room temperature. A four-fold excess of lithium bromide (TCI America) dissolved in 2-butanone (Sigma) was added to the reaction mixture and heated at 50 °C for 10 min, forming a precipitate. The precipitate was washed several times in 2-butanone, dried overnight, and stored at 4 °C under nitrogen in the dark until use. The structure was confirmed by ^1^H NMR (Bruker, Supplementary Fig. 14).

### Peptide synthesis

The α-SMA blocking peptide (Ac-EEEDGRQIKIWFPNRRMKWKK)^31^,^42^ was synthesized using standard solid supported Fmoc protected methods. The peptide was cleaved in trifluoroacetic acid under vigorous stirring for 4 h, precipitated in cold ether, dissolved in water, frozen at −80 °C, and lyophilized. Successful synthesis was confirmed by MALDI (Supplementary Fig. 15).

### *In situ* stiffening

MeHA hydrogels were stiffened at a user-defined time point either before or during cell culture. Soft hydrogels were incubated in culture media containing 6.6 mM LAP photoinitiator for 30 min at 37 °C followed by blue light irradiation for 5 min at 10 mW cm^−2^ using a dental curing lamp (ESPE Elipar 2500, 3 M). Following light exposure, hydrogels were rinsed twice in PBS to remove LAP and placed in fresh culture media.

### Hydrogel mechanical characterization

Hydrogel mechanics were assessed using rheology and atomic force microscopy (AFM). Rheometer experiments were performed on an AR2000ex (TA Instruments) with a light guide connected to the blue light lamp. MeHA hydrogels were allowed to form via an initial addition reaction at pH 10 for 30 min and then were exposed to blue light for 15 min (3 mW cm^−2^). The elastic moduli of hydrogel thin films were obtained from AFM (Agilent 6000ILM). Force curves of 2 × 2 arrays were taken on at least 3 different areas per hydrogel with a 1 μm SiO_2_ spherical probe (0.06 N/m, Novascan). Elastic moduli were obtained from force curve data using the Sneddon approximation of the Hertz indentation model.

### Hepatic stellate cell isolation

Hepatic stellate cells were isolated from Sprague-Dawley rats as previously described^43^. Rat livers were enzymatically digested *in situ* by treatment with 0.4% pronase (Roche Diagnostics) and 0.04% collagenase II (Worthington). The liver slurry was diluted in minimal essential media (MEM) and filtered through cheesecloth. The cell suspension was then washed twice in 0.002% DNase (Worthington). Stellate cells were isolated by density gradient centrifugation with a 9% Nycodenz (Sigma) solution at 1400 g for 25 min, washed in MEM, and stored on ice until seeding on hydrogels.

### Hepatic stellate cell culture on MeHA hydrogels

Prior to cell seeding, hydrogel thin films were incubated in PBS overnight at 37 °C and then sterilized under germicidal UV irradiation for 2 h. Sterilized hydrogels were then incubated in culture media for at least 30 min prior to seeding. Culture media consisted of phenol red-free M199 media (Invitrogen) supplemented with 10 v/v% fetal bovine serum (Sigma), 2 v/v% penicillin streptomycin (Invitrogen), and 1 v/v% fungizone amphotericin B (Invitrogen). Cells were seeded on hydrogel thin films in 6-well plates at a density of 5 × 10^3^ cells/cm^2^ for all experiments except for α-SMA inhibition experiments, which were seeded at 3.5 × 103 cells/cm^2^. Media was replaced 2–3 times per week. For α-SMA inhibition studies, culture media was supplemented with α-SMA blocking peptide (25 μg/mL) and changed daily.

### Time-lapse microscopy

Time-lapse microscopy (Olympus IX81 equipped with environmental chamber for live cell imaging) was used to track stellate cell spreading in real-time following hydrogel stiffening. Images of stellate cells cultured on soft and stiff static substrates, as well as *in situ* stiffened hydrogels were captured every 15 min for 24 h.

### Cell imaging, staining, and quantification

Phase contrast images of stellate cells on hydrogels were acquired using a Zeiss Axiovert 200 inverted microscope (Hitech Instruments, Inc.). NIH ImageJ was used to measure cell spread areas. Hydrogels were prepared for immunocytochemistry by fixing cells in 10% buffered formalin for 15 min, permeabilizing in 0.1% Triton X-100 for 15 min, and blocking in 3% BSA in PBS for 1 h at room temperature. Cell-hydrogel constructs were then incubated with primary antibodies diluted in blocking buffer against α-SMA (mouse monoclonal anti−α-SMA clone 1A4 Ab, Sigma, 1:400), YAP (rabbit polyclonal anti-YAP, Santa Cruz Biotechnology, 1:200), p53 (mouse monoclonal anti-p53, Santa Cruz Biotechnology, 1:400), β-PDGFR (rabbit monoclonal anti-PDGF receptor beta, Cell Signaling Technology, 1:200), and/or β1 integrin (rabbit monoclonal anti-integrin beta 1, Abcam, 1:200) overnight at 4 °C. For staining of the cell surface proteins β-PDGFR and β1 integrin the permeabilization step was omitted. Hydrogels were washed three times in PBS and then incubated with appropriate secondary antibodies (AlexaFluor^®^ 488 goat anti-mouse IgG or AlexaFluor^®^ 568 goat anti-rabbit, Invitrogen) or rhodamine phalloidin to visualize F-actin (Invitrogen, 1:200) for 2 h at room temperature. Hydrogels were then washed twice with PBS, incubated with DAPI for 1 min (1:10000), rinsed again in PBS, and stored at 4 °C in the dark until imaging. Fluorescent images were acquired using an Olympus BX51 microscope (B&B Microscopes Limited). YAP/TAZ nuclear and cytoplasmic intensities as well as relative β-PDGFR and β1 staining intensity were measured in NIH ImageJ.

### Quantitative PCR

RNA was extracted from stellate cells on hydrogels using an RNeasy Plus Micro kit (Qiagen). RNA was reverse transcribed to cDNA using Super Script III Reverse Transcriptase (Invitrogen). Quantitative PCR was performed with a StepOnePlus instrument (Applied Biosystems) for targets including β1 integrin (*Itgb1*), β-PDGFR (*Pdgfrb*), α-SMA (*Acta2*), and type I collagen (*Col1a1*). Ribosomal protein S12 (*Rps12*) and 18S ribosomal RNA (*18S*) were used for normalization. The sequences of the primers are presented in Supplementary Table 1.

### Traction force microscopy

Traction forces exerted on hydrogels by hepatic stellate cells were measured as described previously^21^. Hydrogels embedded with fluorescent microspheres were fabricated as described earlier and fixed to culture plates with a UV-curable fixative (NOA68, Norland Products). Phase contrast and fluorescent images of multiple cells and embedded beads were captured at 20× magnification on a DeltaVision Deconvolution Microscope (GE Healthcare Life Sciences). Image sequences for each cell were taken before and after cell lysis with sodium dodecyl sulfate (SDS) buffer. All imaging was performed in an environmental chamber (37 °C, 5% CO_2_). Data analysis (stack alignment, particle image velocitometry, and Fourier transform traction cytometry) was performed using a freely available plugin suite for ImageJ, created by Tseng *et al.*^44^, which was adapted from Dembo and Wang^45^. For Fourier transform traction cytometry, the Poisson’s ratio of the hydrogel was assumed to be 0.45 and a regularization parameter of 10^−9^ was used. Traction force vector maps were analyzed to determine the average force generation by each cell using a custom MATLAB script.

### Statistical analysis

Student’s t-tests (data sets with only two groups) or one-way analysis of variance (ANOVA) followed by Tukey-HSD post-hoc tests (data sets with more than two groups) were performed. Significance was set at P < 0.05 with *^,^**, or *** indicating P < 0.05, 0.01, or 0.001 respectively. Error is reported in figures as the standard error of the mean unless otherwise noted.

## Additional Information

**How to cite this article**: Caliari, S. R. *et al.* Stiffening hydrogels for investigating the dynamics of hepatic stellate cell mechanotransduction during myofibroblast activation. *Sci. Rep.* **6**, 21387; doi: 10.1038/srep21387 (2016).

## Supplementary Material

Supplementary Movie S1

Supplementary Information
