# Supplementary material for: Stiffening hydrogels for investigating the dynamics of hepatic stellate cell mechanotransduction during myofibroblast activation
Source: Sci Rep. 2016 Feb 24;6:21387. doi: 10.1038/srep21387 (PMC4764908; doi:10.1038/srep21387)
Supplement: Supplementary Information [file srep21387-s2.doc]

**Stiffening hydrogels for investigating the dynamics of hepatic stellate cell mechanotransduction during myofibroblast activation**

Steven R. Caliari^1^, Maryna Perepelyuk^2^, Brian D. Cosgrove^1^, Shannon J. Tsai^2^, Gi Yun Lee^1^, Robert L. Mauck^1,3^, Rebecca G. Wells^2^, Jason A. Burdick^1*^

^1^ Department of Bioengineering

^2^ Department of Medicine

^3^ Department of Orthopedic Surgery

University of Pennsylvania

Philadelphia, PA 19104

***Corresponding Author:**

J.A. Burdick

Department of Bioengineering

University of Pennsylvania

240 Skirkanich Hall

210 S. 33^rd^ St.

Philadelphia, PA 19104

Phone: (215) 898-8537

Fax: (215) 573-2071

email: burdick2@seas.upenn.edu

**Supplementary Information**

**Supplementary Information Figures**

**
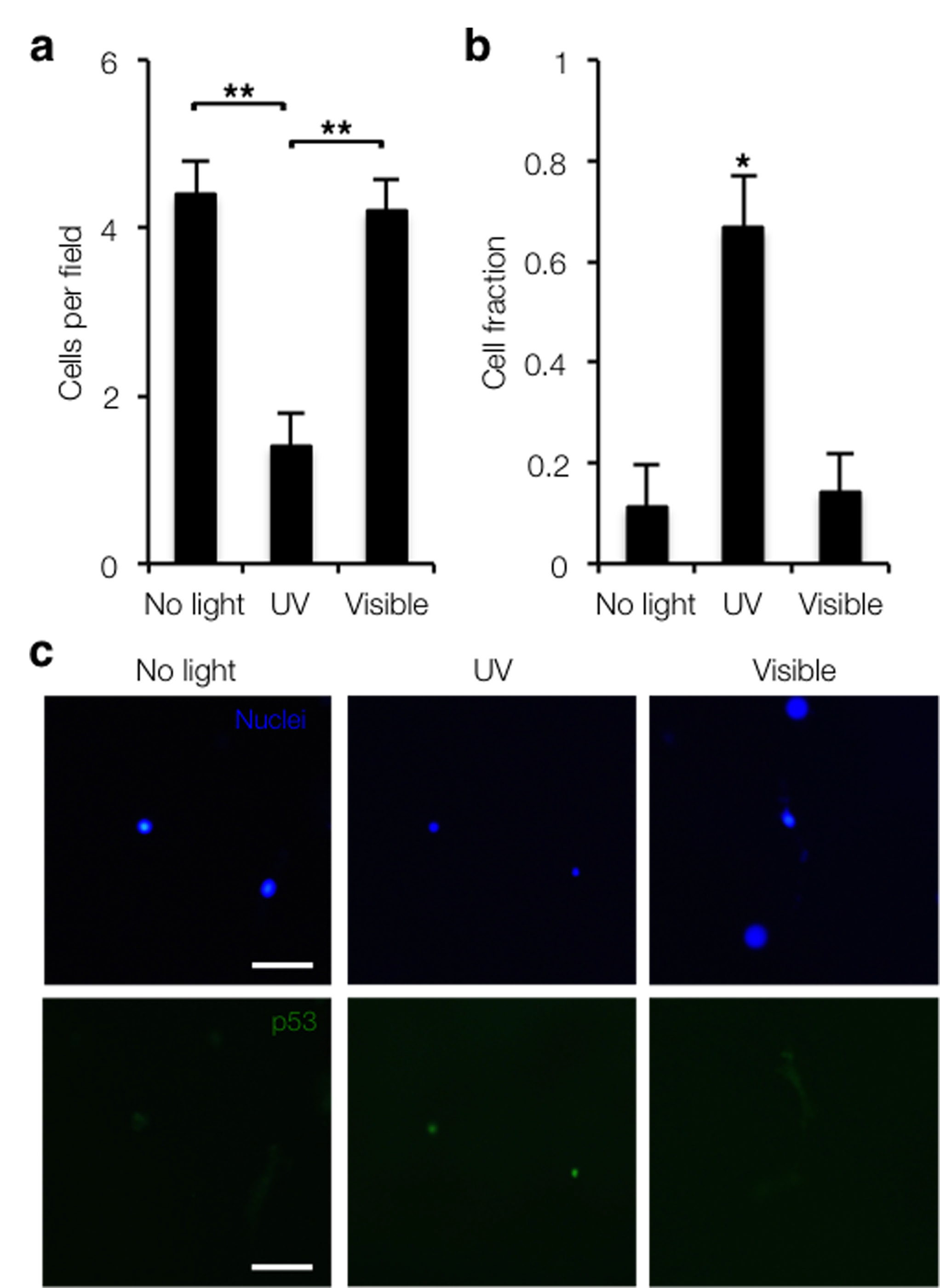
**

**Supplementary Fig. 1. UV irradiation leads to reduced stellate cell viability and p53 nuclear accumulation.** (**a**) Stellate cells were cultured on soft gels for 3 days, exposed to light (UV: 2 min, 10 mW/cm^2^; visible: 5 min, 10 mW/cm^2^), and then assessed for viability 24 h later. UV irradiation alone led to reduced viability. Cell counts were taken from 5 separate fields of view, error bars represent s.e.m. (**b**) Cells exposed to UV light showed increased nuclear p53 staining. (*n* > 21 cells per group, error bars represent s.e.m.) (**c**) Representative p53 immunostaining. Scale bars: 50 μm. *: *P* < 0.05, **: *P* < 0.01.

**
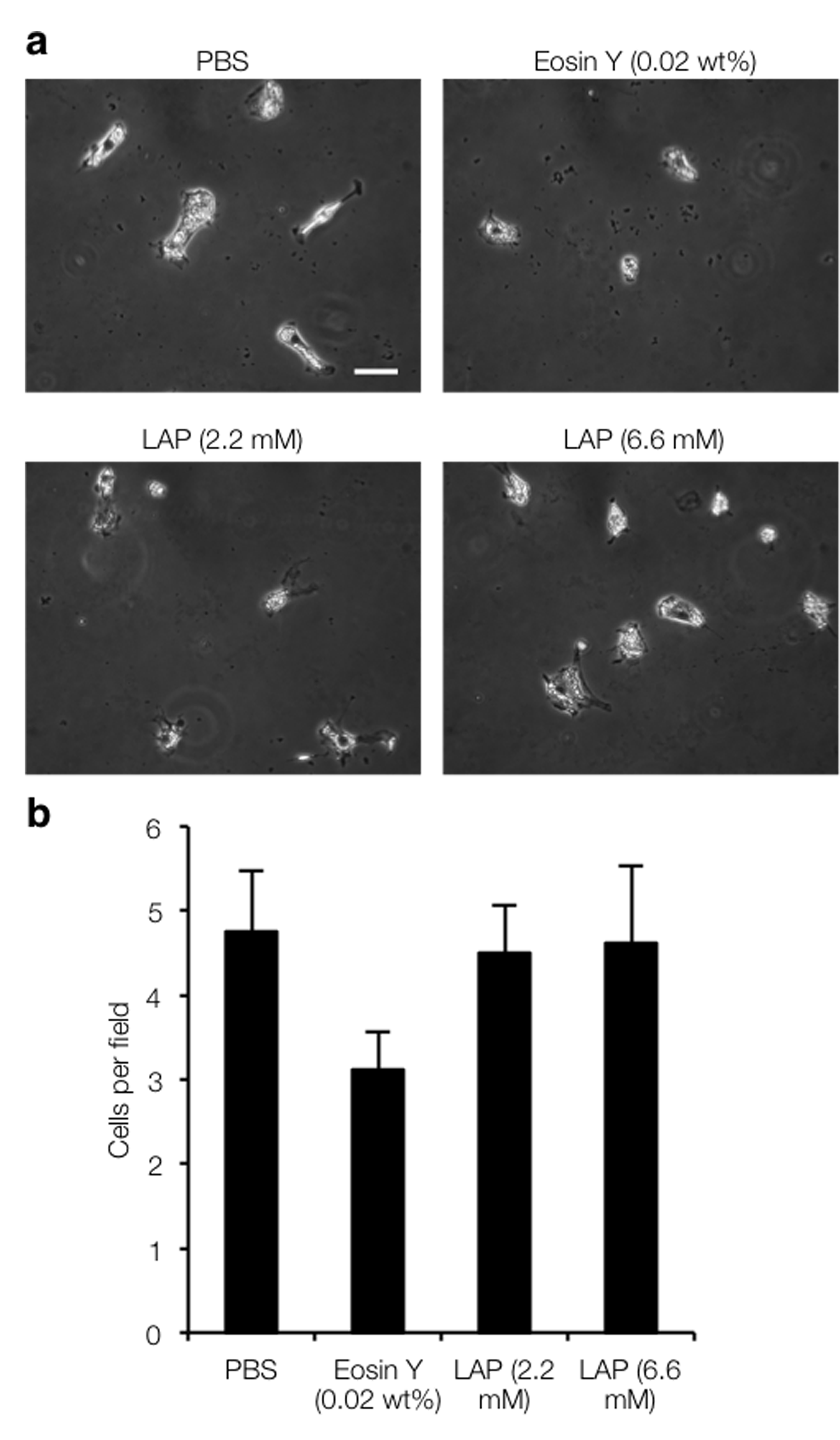
**

**Supplementary Fig. 2. LAP is not cytotoxic to stellate cells.** (**a**) Stellate cells were seeded on soft gels, cultured for 3 days, exposed to cell culture media supplemented with initiator (or PBS as control) for 30 min at 37°C, rinsed with PBS twice, and then assayed for viability 24 h later. Representative phase contrast images showed that while the common visible light photoinitiator eosin Y reduced cell viability, LAP showed no detrimental effects even at high concentrations (6.6 mM). Scale bar: 50 μm. (**b**) Quantification of cell survival after 24 h. Cell counts were taken from eight separate fields of view (error bars represent s.e.m).

**
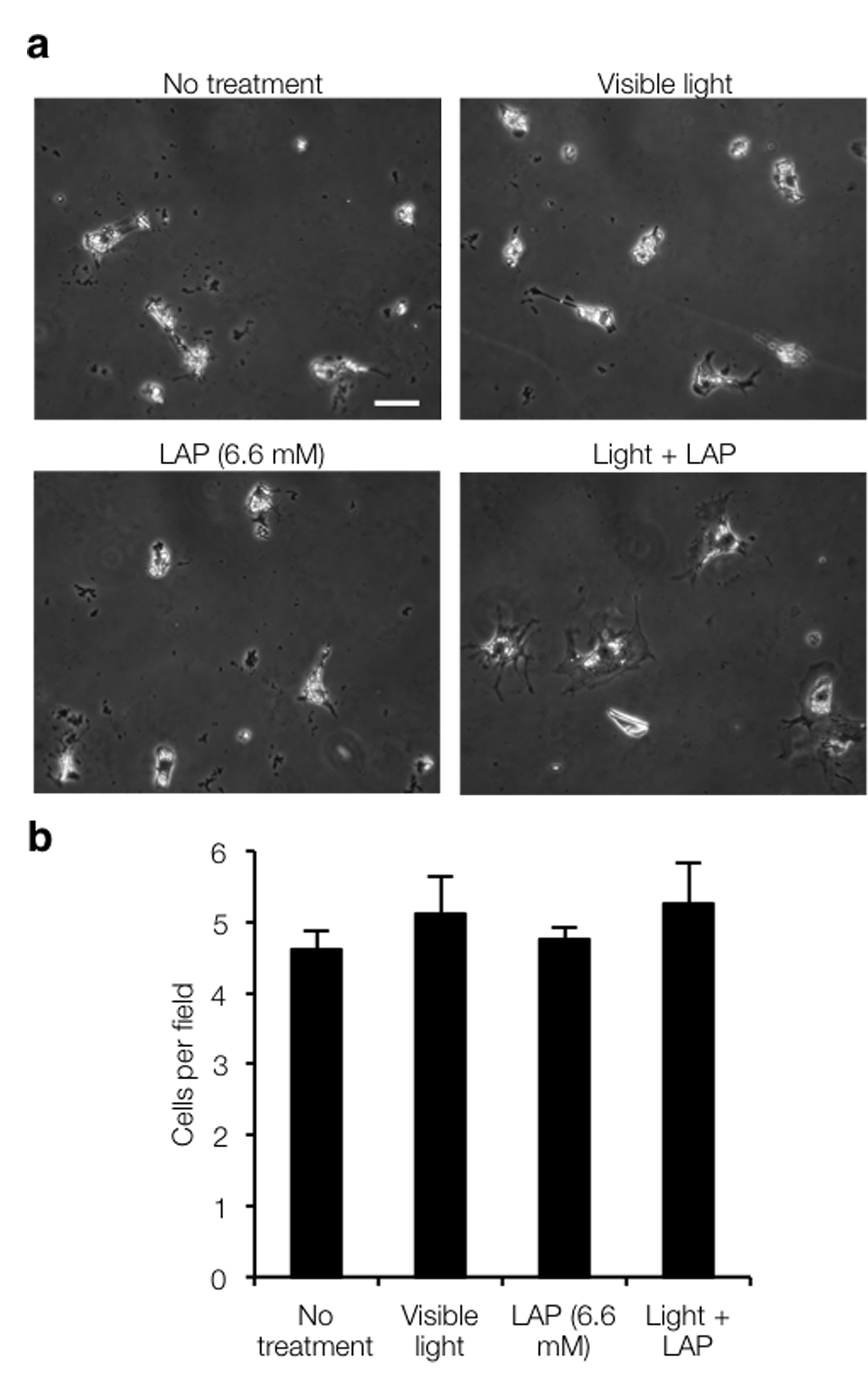
**

**Supplementary Fig. 3. Visible light and LAP are not cytotoxic to stellate cells.** (**a**) Stellate cells were plated on soft gels, cultured for 3 days, and exposed to visible light alone (5 min, 10 mW/cm^2^), initiator alone (LAP, 6.6 mM), or both light and initiator. Representative phase contrast images to assess cell viability 24 h later showed no detrimental effects of light or initiator exposure. Scale bar: 50 μm. (**b**) Cell counts for each experimental group, averaged from eight separate fields of view (error bars represent s.e.m.).

**
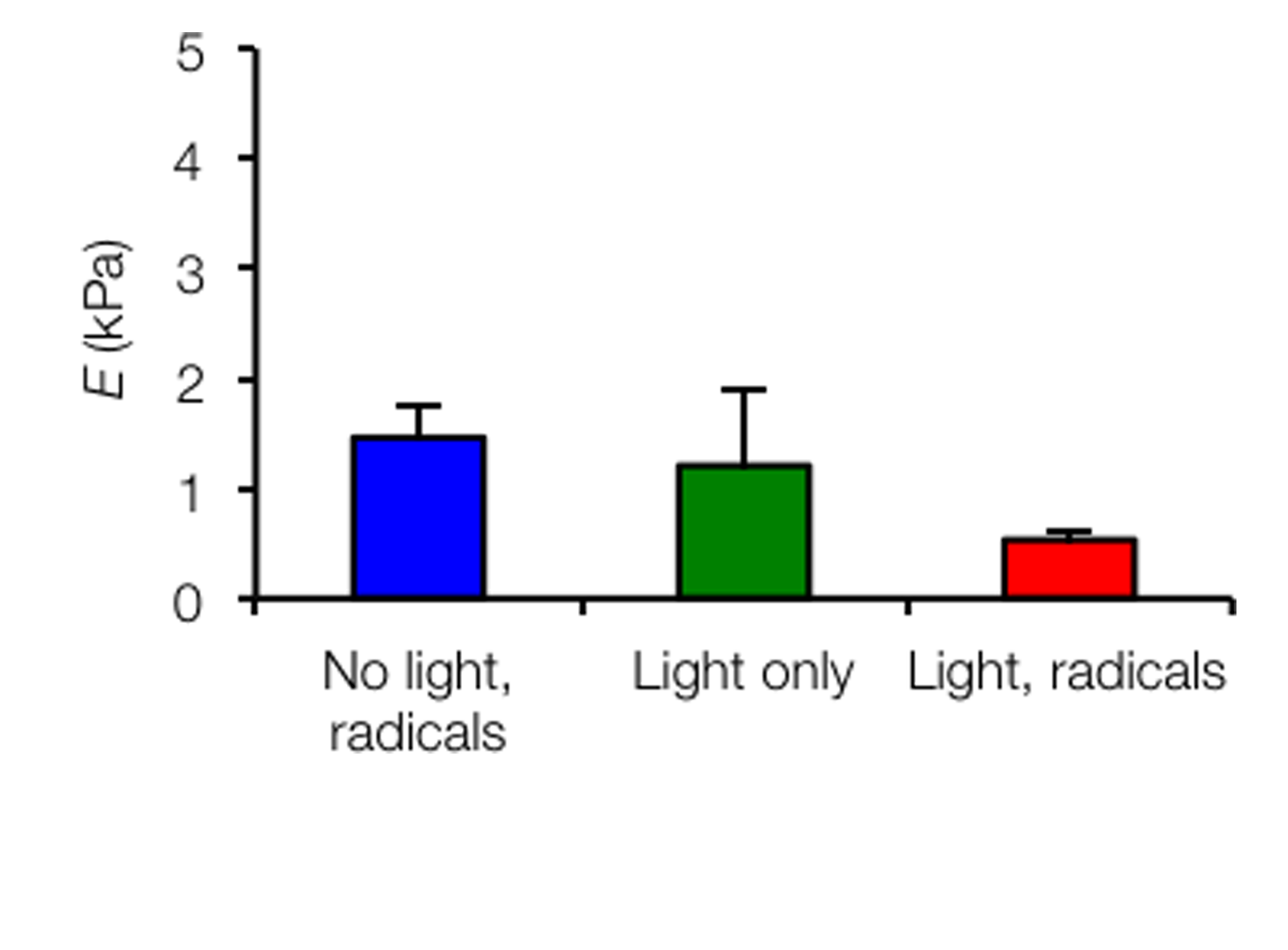
**

**Supplementary Fig. 4. Mechanical testing of thiol-terminated soft MeHA gels demonstrates no increase in stiffness in response to free radical generation.** (*n* = 3 gels per group).

**
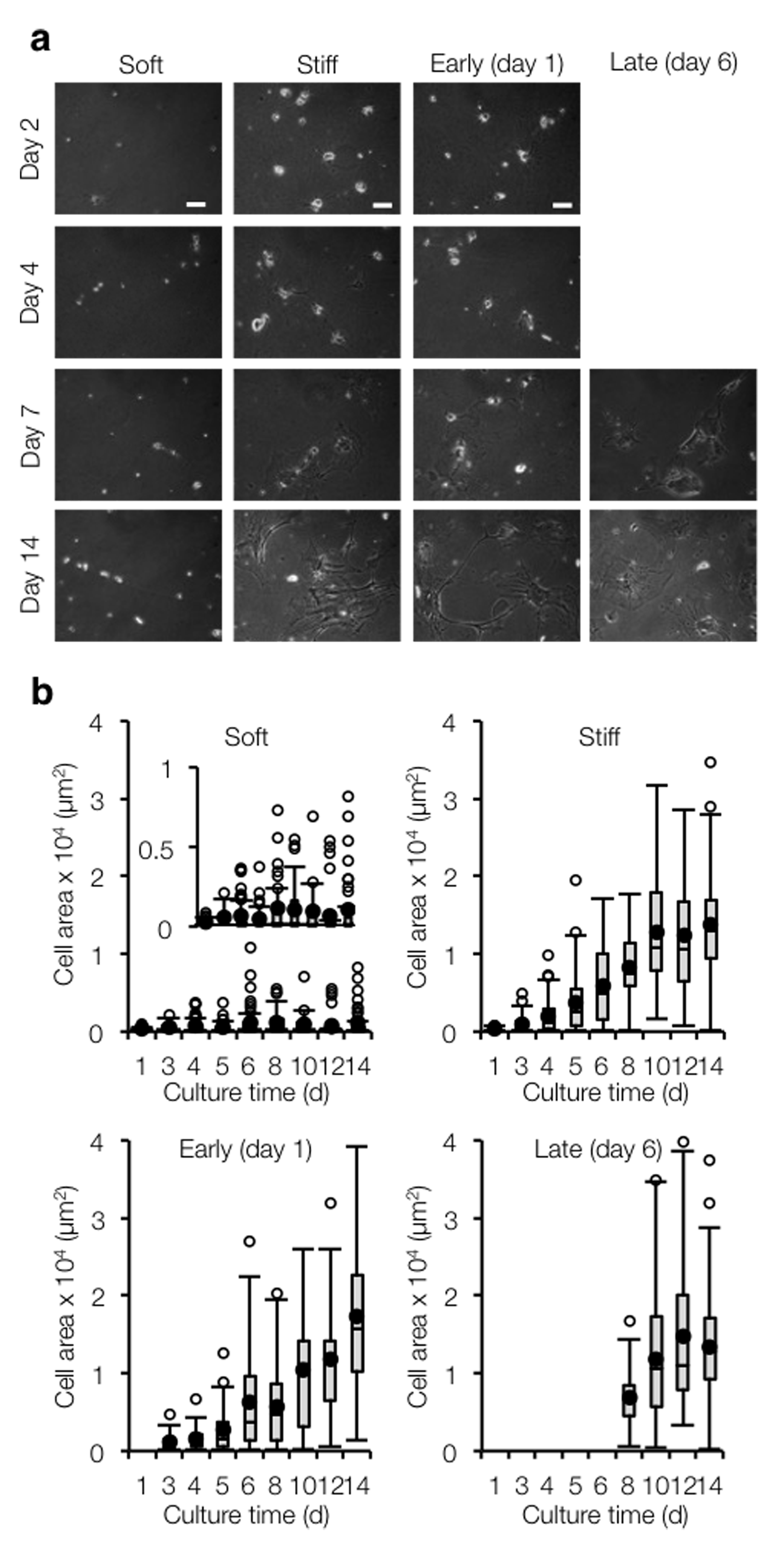
**

**Supplementary Fig. 5. Hepatic stellate cells spread more rapidly following later stiffening.** (**a**) Representative phase contrast images of stellate cells on soft, stiff, early (day 1), and late (day 6) hydrogels. Scale bars: 50 μm. (**b**) Tukey box plot quantification of stellate cell spread areas over the course of 14 days (*n* > 25 cells per group per time point).

**
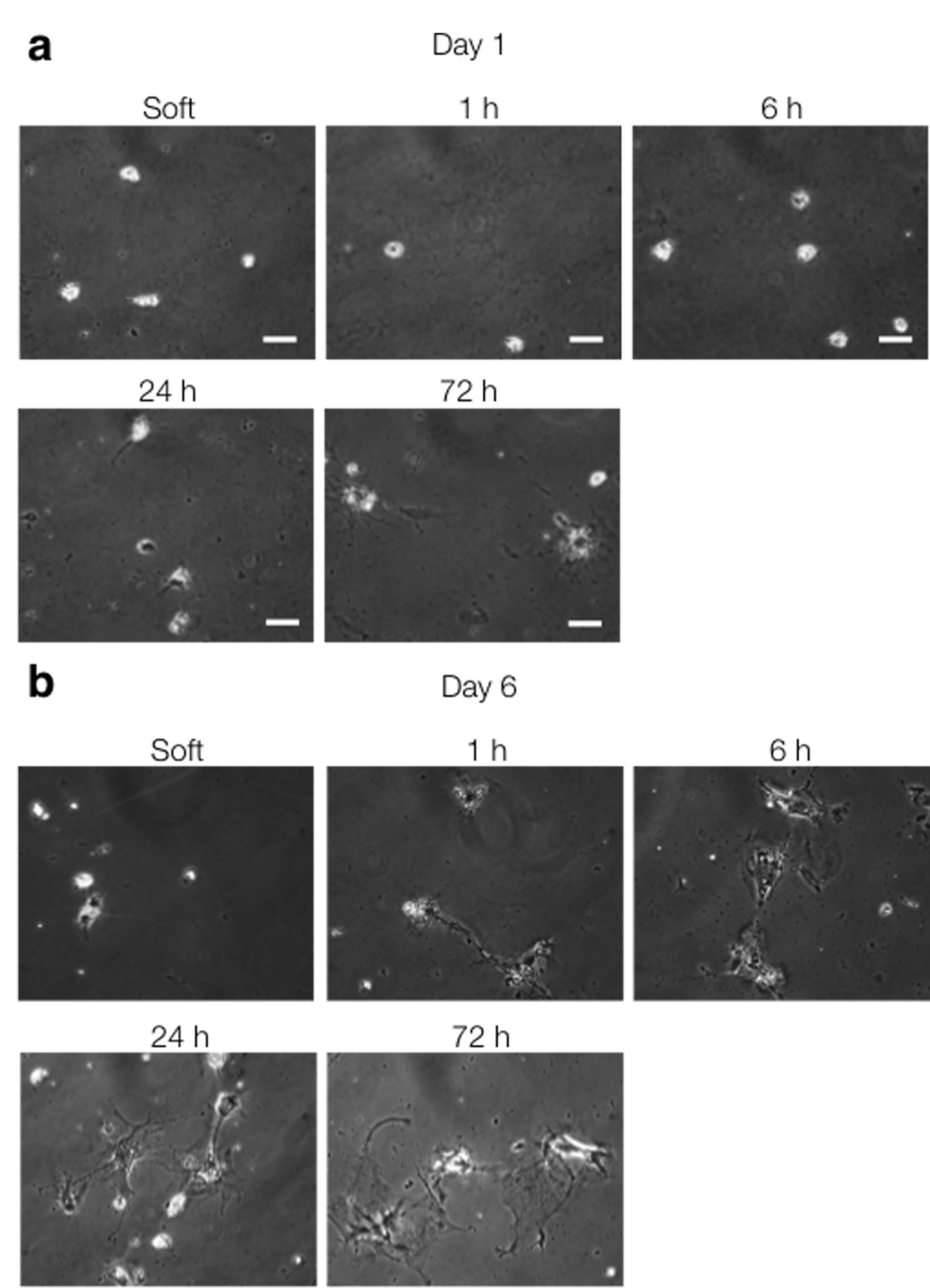
**

**Supplementary Fig. 6. Representative set of phase contrast images of stellate cells on hydrogels from Figure 3.** (**a**) Day 1 (early stiffening). (**b**) Day 6 (late stiffening). Cell images were taken 1, 6, 24, or 72 h following stiffening. Scale bars: 50 μm.

**
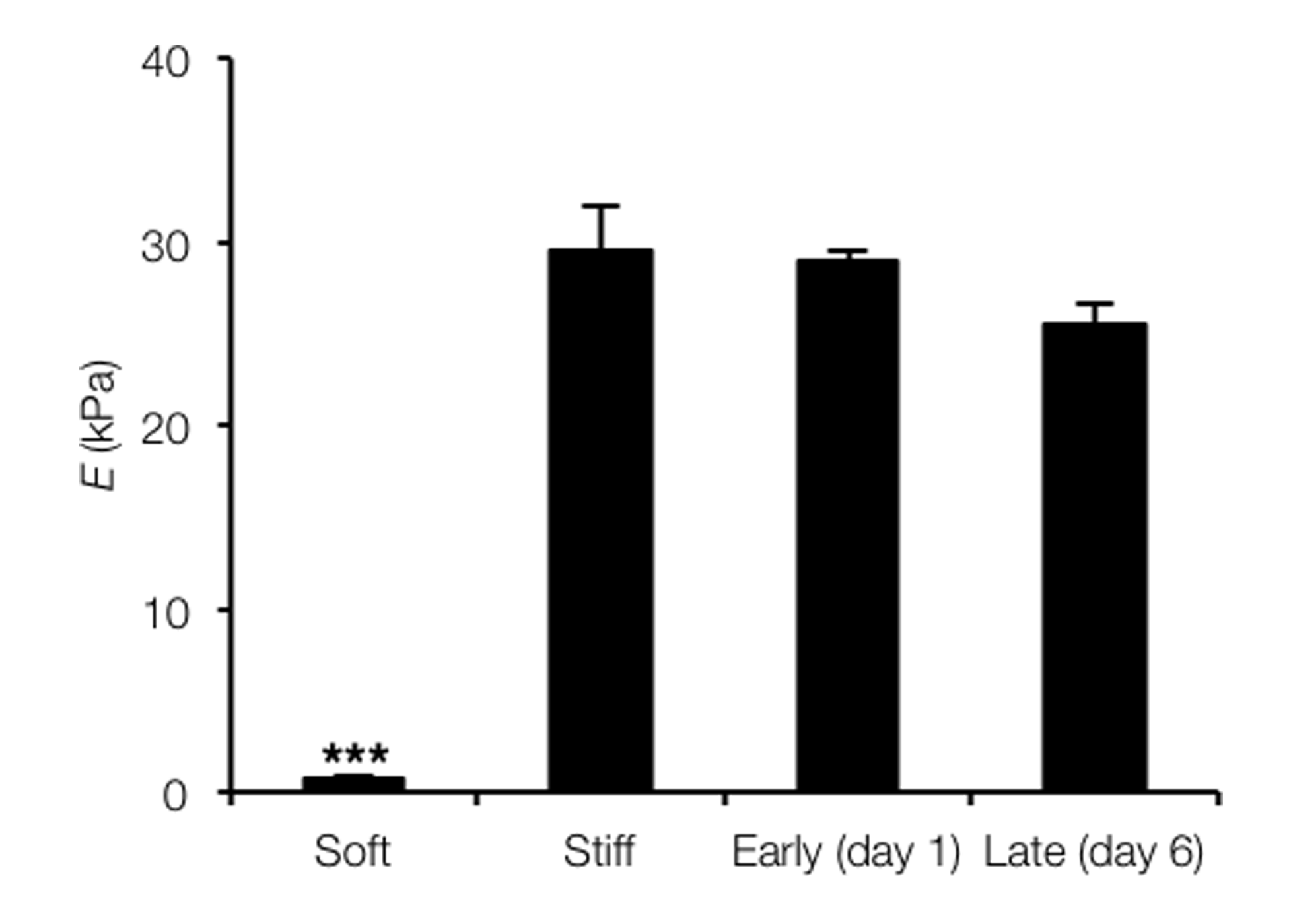
**

**Supplementary Fig. 7. MeHA hydrogel stiffness is not affected by stiffening time point.** AFM measurements of hydrogel stiffness after 14 days incubation in cell culture media at 37°C showed no significant differences between groups stiffened at different time points (*n* = 3 gels per group). ***: *P* < 0.001 compared to stiffened groups.

**
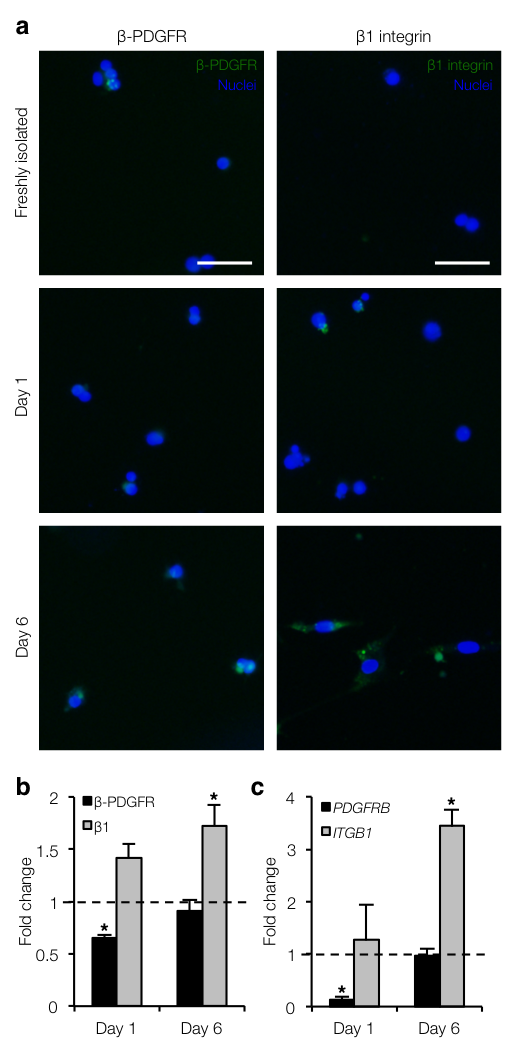
**

**Supplementary Fig. 8. Stellate cell surface protein expression recovers with culture on soft hydrogels.** (**a**) Stellate cells cultured on soft gels for 6 days display increased expression of surface proteins β-PDGFR and β1 integrin. Scale bars: 50 μm. (**b**) Relative staining intensity for β-PDGFR and β1 integrin (normalized to DAPI stain, dashed line represents levels for freshly isolated stellate cells). (**c**) Expression of the genes encoding for β-PDGFR (*PDGFRB*) and β1 integrin (*ITGB1*) was assessed for freshly isolated stellate cells (dashed line) and compared to profiles for cells cultured for 1 or 6 days on soft hydrogels (*n* = 3 with each sample containing pooled RNA from 5-7 replicate gels, error bars represent s.e.m.) *: *P* < 0.05 compared to freshly isolated stellate cells.

**
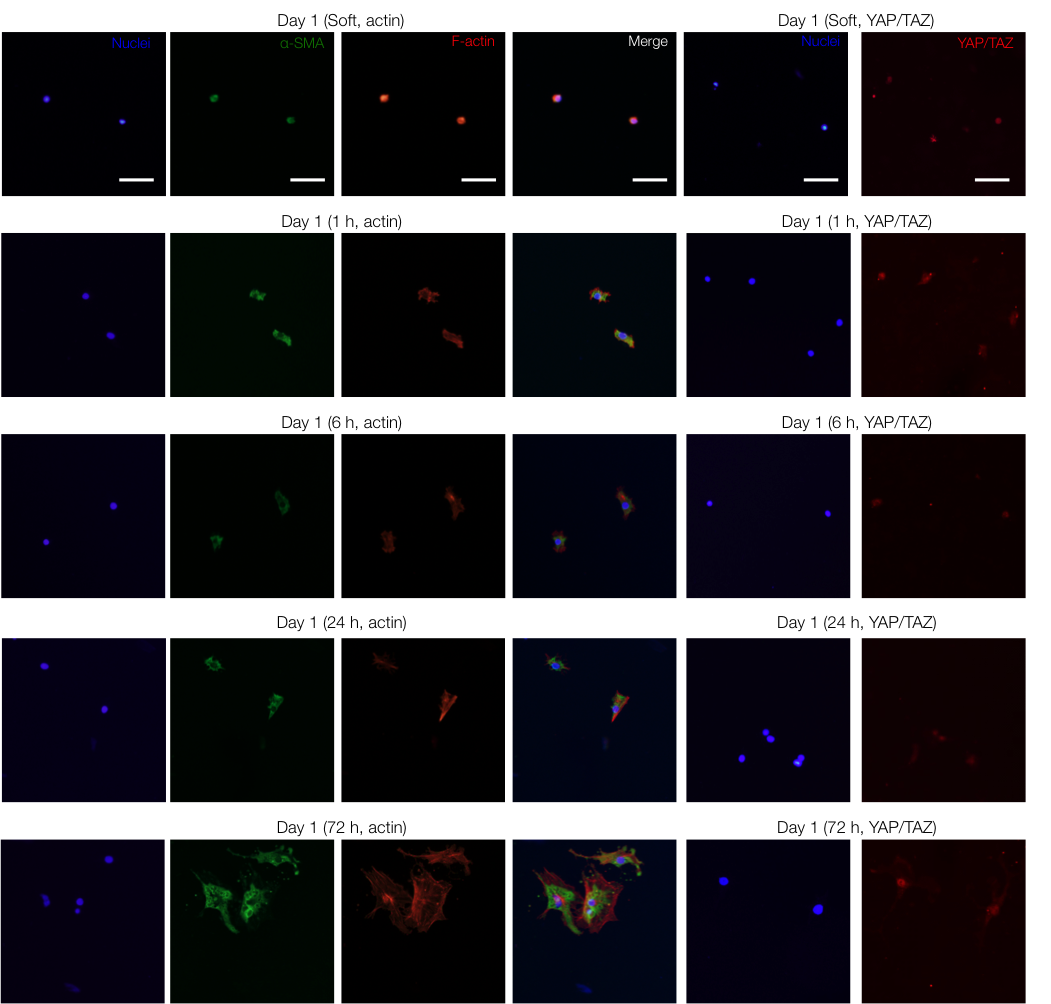
**

**Supplementary Fig. 9. Representative immunostaining for data presented in Figure 3 (Day 1).** Images for 1 h and 72 h time points are replicated from Figure 3 for comparison. Scale bars: 50 μm.

**
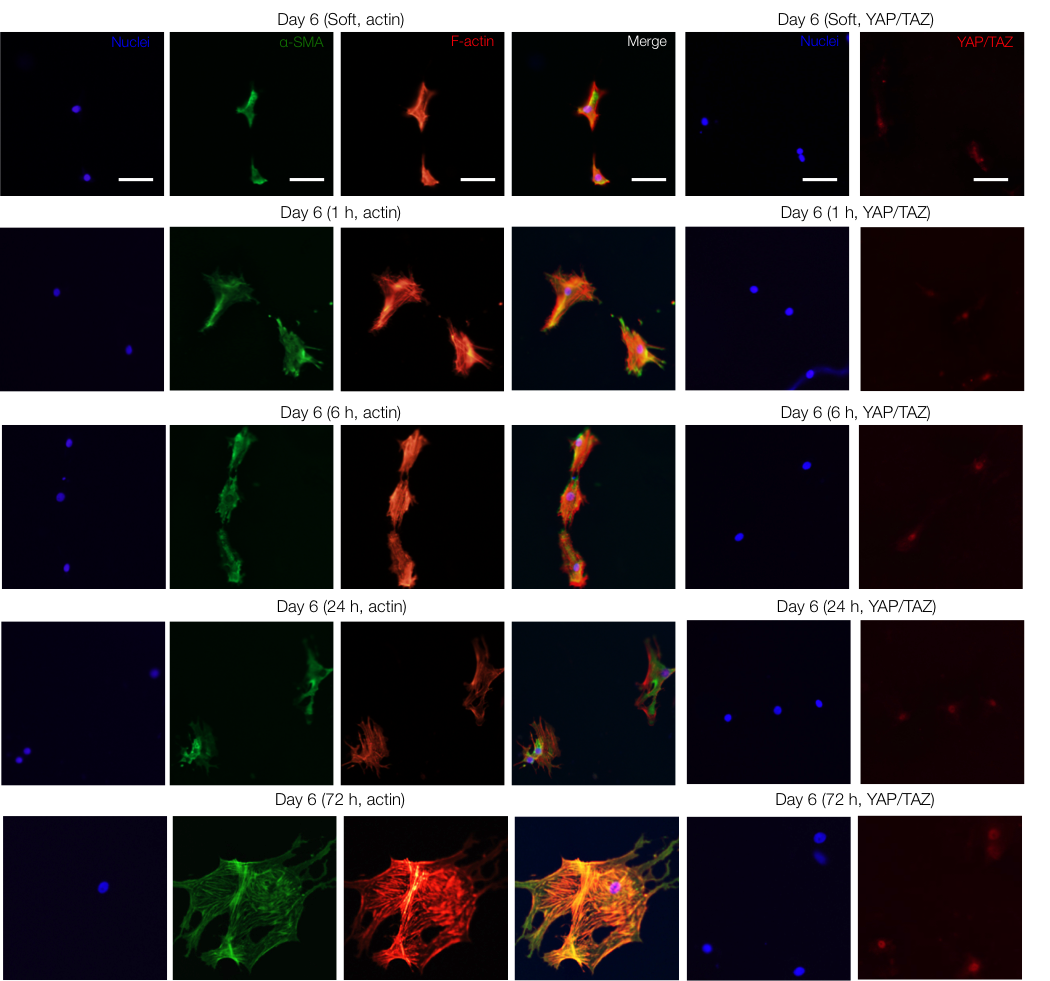
**

**Supplementary Fig. 10. Representative immunostaining for data presented in Figure 3 (Day 6).** Images for 1 h and 72 h time points are replicated from Figure 3 for comparison. Scale bars: 50 μm.


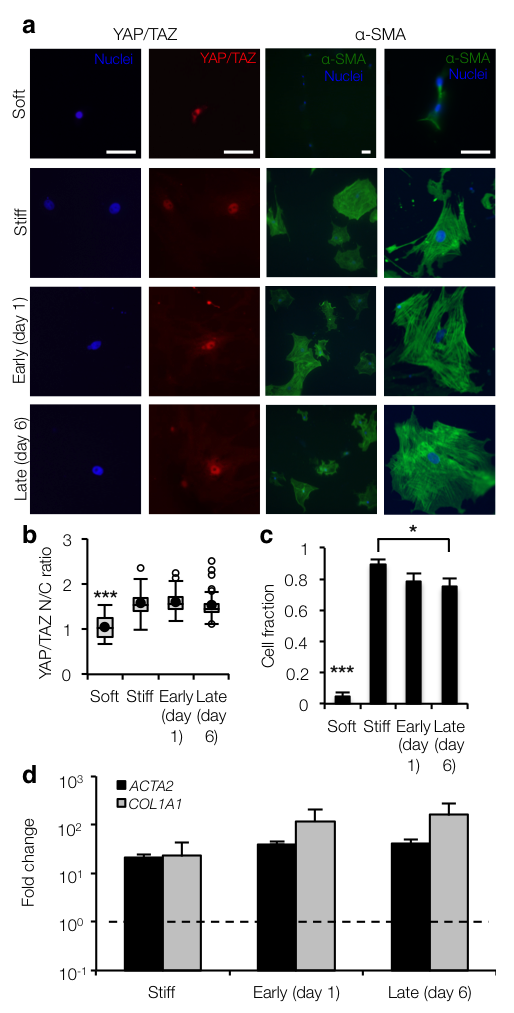


# Supplementary Fig. 11. Stellate cells display similar myofibroblast phenotype independent of stiffening time point after 14 days. (a) Following 14 days, the majority of stellate cells cultured on stiff substrates showed increased YAP/TAZ nuclear translocation and organized α-SMA stress fibers regardless of stiffening time point while stellate cells on soft gels show reduced YAP/TAZ nuclear localization and only diffuse α-SMA staining. Scale bars: 50 μm. (b) Tukey box plot quantification of YAP/TAZ nuclear to cytoplasmic intensity ratio demonstrated significantly higher levels of nuclear intensity in stiffened groups compared to soft group (*n >* 22 cells per group). (c) Quantification of cell fraction displaying organized α-SMA stress fibers (*n* > 43 cells per group, error bars represent s.e.m.). (d) Up-regulation of fibrogenic genes *ACTA2* (α-SMA) and *COL1A1* (type I collagen) was observed compared to quiescent cells (*n* = 3 with each sample containing pooled RNA from 6 replicate gels, error bars represent s.e.m.). *: *P* < 0.05; ***, *P* < 0.001.

**
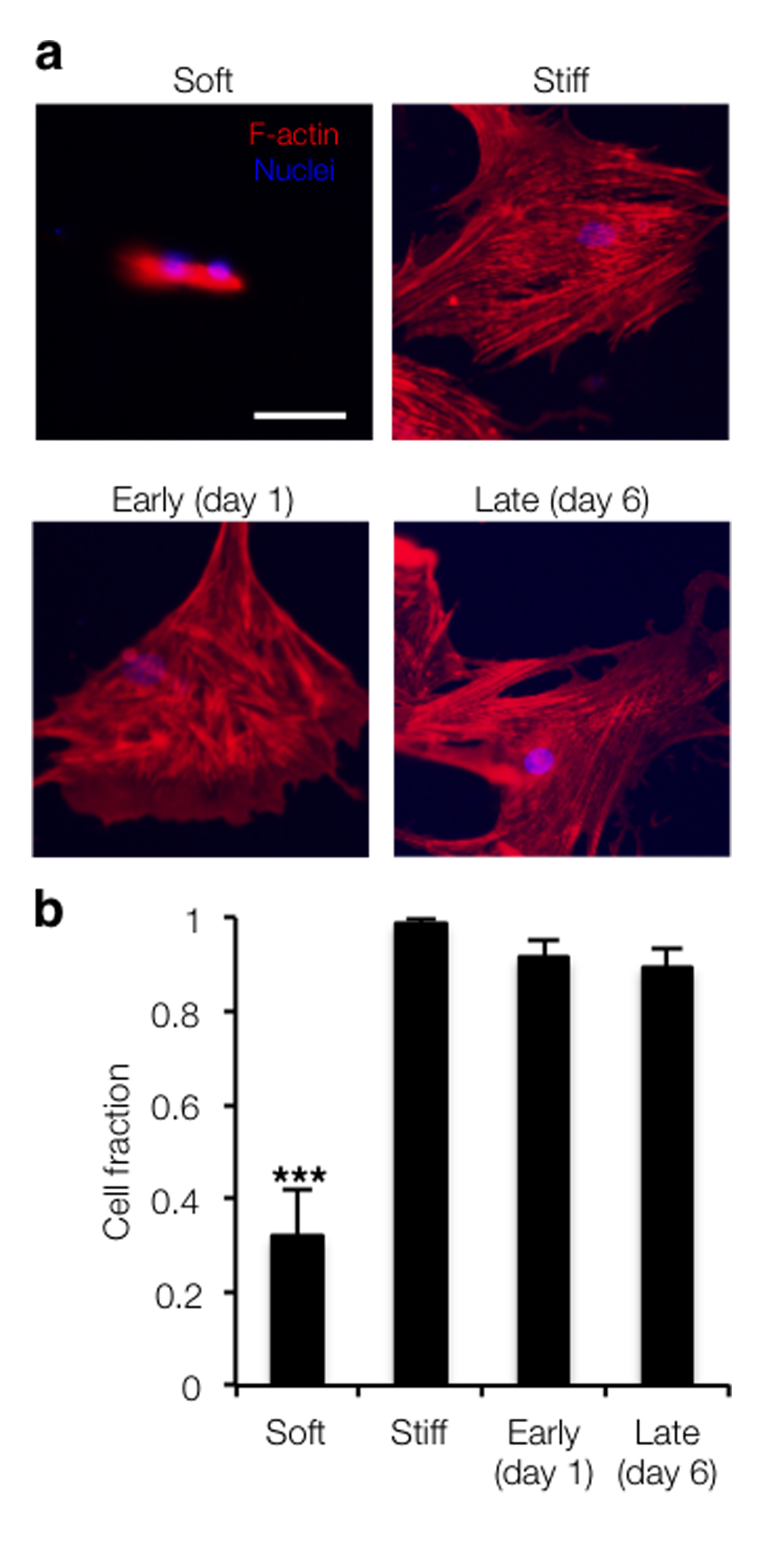
**

**Supplementary Fig. 12. Day 14 F-actin immunostaining.** (**a**) After 14 days of culture nearly all stellate cells on stiffened substrates display F-actin stress fibers. Representative images shown for soft, stiff, early (day 1), and late (day 6) groups. Scale bar: 50 μm. (**b**) Quantification of stellate cell fraction displaying F-actin stress fibers (*n* > 44 cells per group, error bars represent s.e.m.). ***: *P* < 0.001 compared to stiffened groups.

**
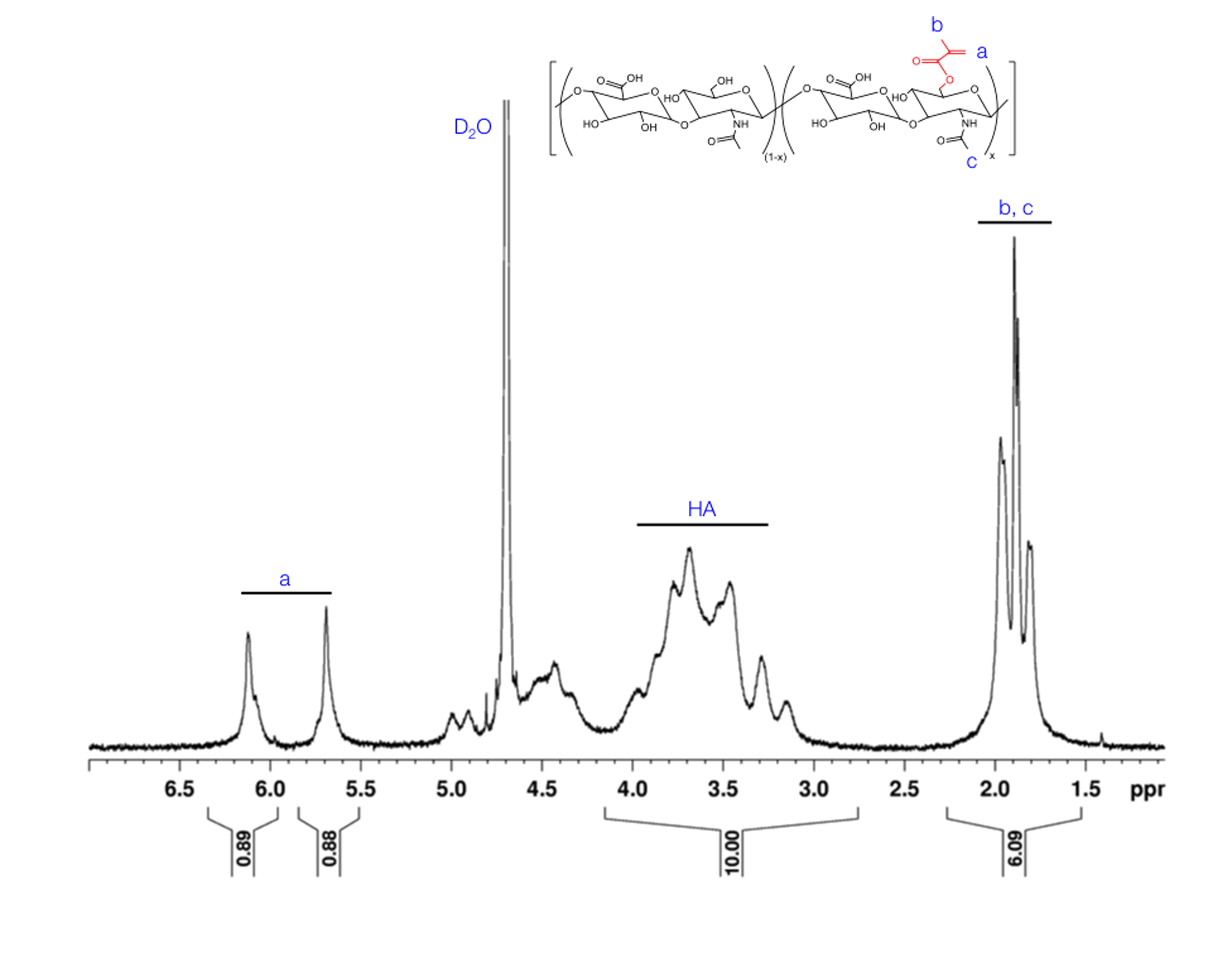
**

**Supplementary Fig. 13. ^1^H NMR spectrum of methacrylated hyaluronic acid (MeHA).** Spectrum indicates ~ 90% modification of HA repeat units with methacrylate groups.

**
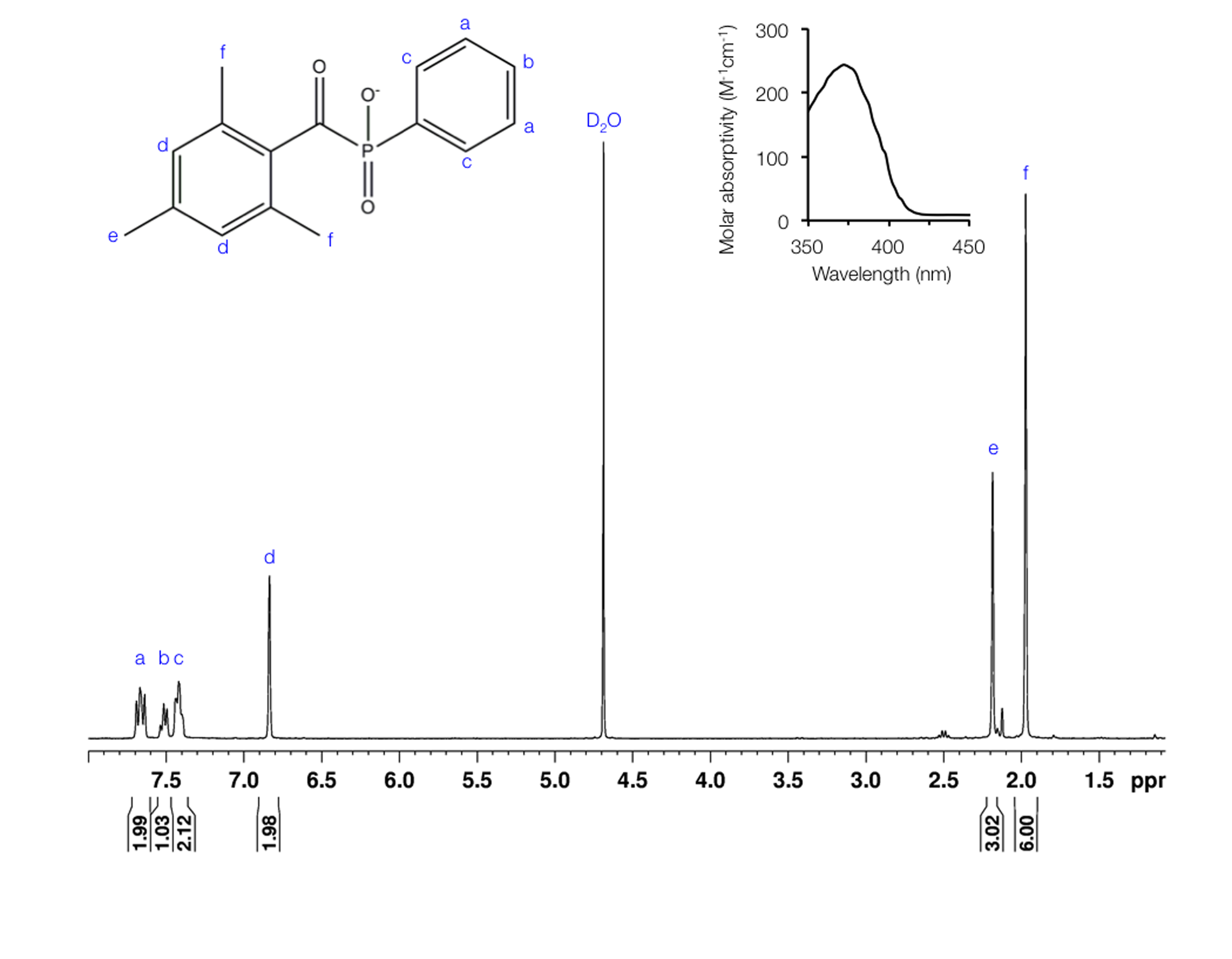
**

**Supplementary Fig. 14.** **^1^H NMR and absorption spectra of lithium acylphosphinate (LAP).** Absorption spectrum shows peak at ~ 370-375 nm with absorption in the visible range (> 400 nm).

**
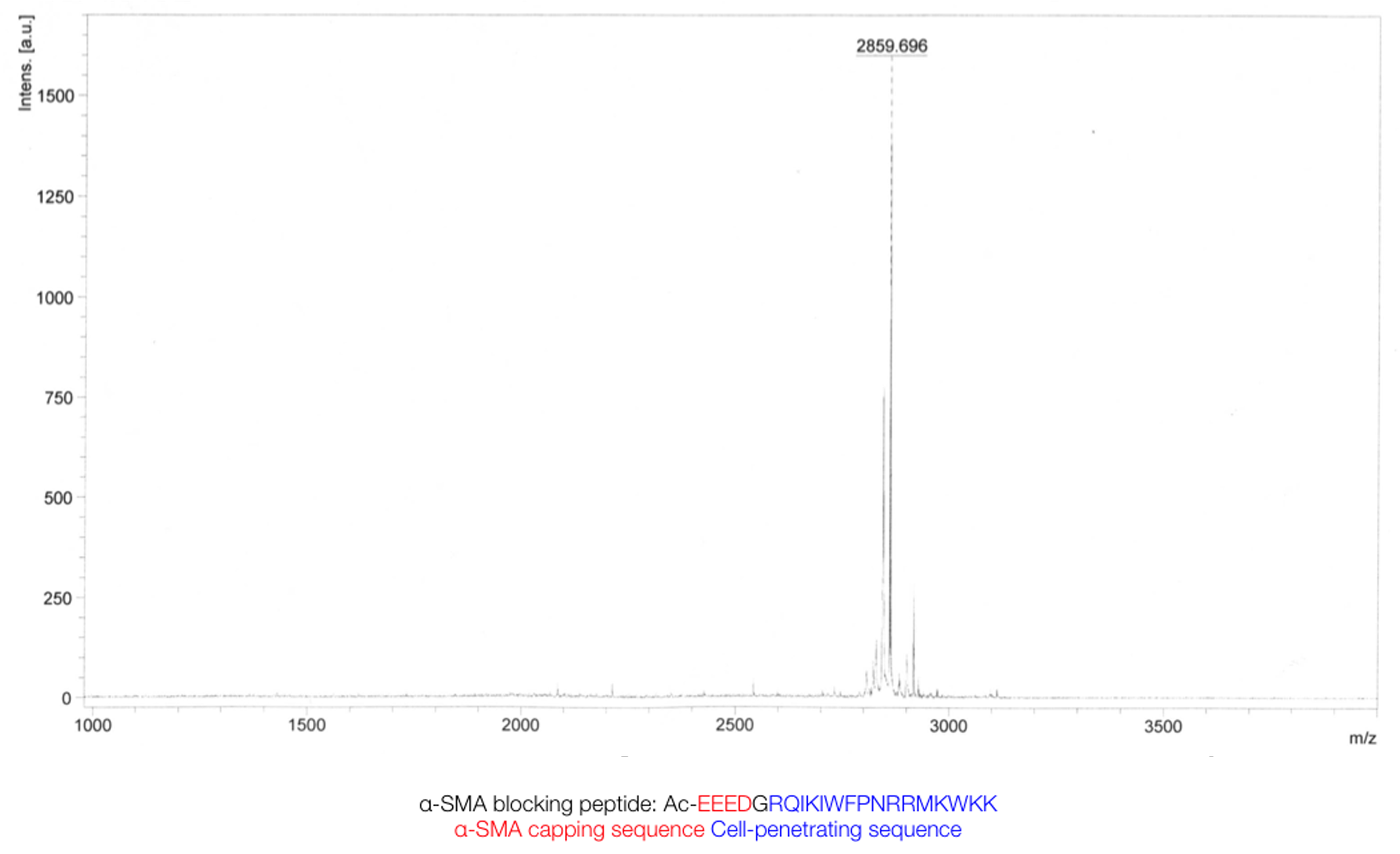
**

**Supplementary Fig. 15. MALDI spectrum of α-SMA blocking peptide.** Expected mass (plus alcohol from resin): 2862 g/mol. Actual mass: 2860 g/mol.

**Supplementary Movie 1. Hepatic stellate cell spreading in response to *in situ* stiffening.** Time lapse of stellate cells spreading in the 24 h following stiffening at day 4. Scale bar: 50 μm.

**Supplementary Information Tables**

| Gene | Primer sequence |
| --- | --- |
| *Pdgfrb* | (F) ATGCAGAAATGCTGGGAAGAA  (R) AAACTCCTCATCCACCTGCTG |
| *Itgb1* | (F) CGCATATCTGGAAACTTGGAC  (R) TTCCCATCTCCAGCAAAGTG |
| *Acta2* | (F) TGTGCTGGACTCTGGAGATG  (R) GAAGGAATAGCCACGCTCAG |
| *Col1a1* | (F) CATAAAGGGTCATCGTGGCT  (R) TTGAGTCCGTCTTTGCCAG |
| *18S* | (F) CCCAGTAAGTGCGGGTCATAA  (R) GATCCGAGGGCCTCACTAAAC |
| *Rps12* | (F) CCTCGATGACATCCTTGG  (R) GGAAGGCATAGCTGCTGG |

**Supplementary Table 1.** **Primers for quantitative PCR used in this study.**
